# Fibrin Stiffness Regulates Phenotypic Plasticity of Metastatic Breast Cancer Cells

**DOI:** 10.1002/adhm.202301137

**Published:** 2023-09-21

**Authors:** Maria Heilala, Arttu Lehtonen, Ossi Arasalo, Aino Peura, Juho Pokki, Olli Ikkala, Nonappa, Juha Klefström, Pauliina M. Munne

**Affiliations:** ^1^ Department of Applied Physics Aalto University P.O. Box 15100 Aalto Espoo FI‐00076 Finland; ^2^ Department of Electrical Engineering and Automation Aalto University P.O. Box 12200 Aalto Espoo FI‐00076 Finland; ^3^ Finnish Cancer Institute and FICAN South Helsinki University Hospital & Cancer Cell Circuitry Laboratory Translational Cancer Medicine Medical Faculty University of Helsinki P.O. Box 63 (Haartmaninkatu 8) Helsinki 00014 Finland; ^4^ Faculty of Engineering and Natural Sciences Tampere University P.O. Box 541 Tampere FI‐33720 Finland

**Keywords:** 3D cell culture, circulating tumor cells, fibrin hydrogels, metastasis, phenotypic plasticity, triple negative breast cancer

## Abstract

The extracellular matrix (ECM)‐regulated phenotypic plasticity is crucial for metastatic progression of triple negative breast cancer (TNBC). While ECM faithful cell‐based models are available for in situ and invasive tumors, such as cell aggregate cultures in reconstituted basement membrane and in collagenous gels, there are no ECM faithful models for metastatic circulating tumor cells (CTCs). Such models are essential to represent the stage of metastasis where clinical relevance and therapeutic opportunities are significant. Here, CTC‐like DU4475 TNBC cells are cultured in mechanically tunable 3D fibrin hydrogels. This is motivated, as in circulation fibrin aids CTC survival by forming a protective coating reducing shear stress and immune cell‐mediated cytotoxicity and promotes several stages of late metastatic processes at the interface between circulation and tissue. This work shows that fibrin hydrogels support DU4475 cell growth, resulting in spheroid formation. Furthermore, increasing fibrin stiffness from 57 to 175 Pa leads to highly motile, actin and tubulin containing cellular protrusions, which are associated with specific cell morphology and gene expression patterns that markedly differ from basement membrane or suspension cultures. Thus, mechanically tunable fibrin gels reveal specific matrix‐based regulation of TNBC cell phenotype and offer scaffolds for CTC‐like cells with better mechano‐biological properties than liquid.

## Graphical abstract

3D fibrin gels are used to model the phenotypic plasticity of circulating tumor cells (CTCs) ‐like breast cancer cells. Compared with standard 3D culture conditions, cells in fibrin gels show unique gene expression signatures. The cell phenotype is subject to regulation by matrix mechanical properties, with stiff fibrin inducing dynamic cell protrusions that resemble prometastatic structures.

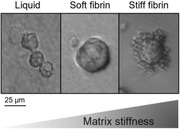

## 1 Introduction

Breast cancer is the most commonly diagnosed cancer type in women and remains the leading cause of cancer‐related death.^[^ ^1^, ^2^ ^]^ The major cause of mortality is the metastatic form of the disease, which is considered incurable.^[^ ^3^ ^]^ More importantly, the intra‐ and intertumoral heterogeneity and the presence of multiple molecular subtypes affects the prognosis, treatment and clinical outcomes in breast cancer.^[^ ^4^ ^]^ Among the breast cancer subtypes, triple negative breast cancer (TNBC) is regarded as the most aggressive form of the disease with a high metastatic tendency and lack of targeted therapies.^[^ ^5^, ^6^ ^]^ An additional challenge stems from the observation that metastatic lesions often exhibit different tumor cell phenotype than the original tumor.^[^ ^7^, ^8^, ^9^, ^10^ ^]^ To find novel treatment modalities for metastasis, it is essential to recognize mechanisms regulating the phenotypic plasticity of tumor cells during the cancer progression.

Metastatic progression can be divided into five main steps, including breach of the basement membrane followed by invasion of the cancerous cells into the surrounding extracellular matrix (ECM), intravasation into blood or lymphatic vessels, survival in circulation, extravasation into distant tissue, and colonization to form a metastatic tumor.^[^ ^11^ ^]^ The number and phenotype of tumor‐derived cells in the bloodstream is associated with breast cancer outcome.^[^ ^12^, ^13^, ^14^ ^]^ Therefore, there is growing interest in utilizing these circulating tumor cells (CTCs) to evaluate disease progression and treatment effectiveness.^[^ ^15^ ^]^ Due to the lack of general marker for metastasis, the metastatic potential of cancer cells is typically predicted by assessing their invasive and migratory behavior.^[^ ^16^ ^]^ Conventional in vitro models for invasion and migration are based on culturing cells in or on matrices that mimic the composition of collagenous ECM or basement membrane extract (BME).^[^ ^16^ ^]^ As breaching of these barriers represents only one part of metastatic progression, other aspects of metastatic processes are poorly recapitulated in these models. Specifically, metastatic spreading is considered a highly inefficient process, with only a small fraction of cancer cells making it into the circulation and establishing secondary tumors.^[^ ^17^ ^]^ To identify factors promoting CTC survival and phenotypic plasticity outside of the primary tumor, complementary in vitro models to traditional invasion and migration assays are urgently needed.

Fibrin(ogen) is one of the ECM components that is known to facilitate metastatic potential of CTCs.^[^ ^18^ ^]^ In vivo, fibrin is generated through the enzymatic cleavage of fibrinogen by thrombin in blood coagulation.^[^ ^19^ ^]^ The physiological function of fibrin is to maintain hemostasis in response to tissue injury. Importantly, activation of coagulation system is also implicated in cancer progression.^[^ ^20^ ^]^ For instance, majority of cancer patients exhibit abnormal blood coagulation profiles, leading to a hypercoagulable state and spontaneous thrombus formation.^[^ ^21^ ^]^ In breast cancer, increased fibrin turnover has been found to correlate with more advanced stages of the disease.^[^ ^22^ ^]^ Furthermore, fibrinogen‐deficiency has been shown to reduce pulmonary metastases in mouse models of lung cancer and melanoma.^[^ ^18^ ^]^ In circulation, fibrin and platelets are proposed to aid tumor cell survival by forming a protective coating that reduces shear stresses and decreases immune cell‐mediated cytotoxicity.^[^ ^23^ ^]^ Additionally, fibrin may promote extravasation into distant tissues by enhancing adhesion to the vessel walls.^[^ ^24^ ^]^ Therefore, fibrin gels are attractive scaffolds to investigate metastatic steps occurring at the interface between circulation and tissue.

Although fibrin gels have been utilized in various tissue engineering and 3D cell culture applications, their use as breast cancer cell culture platforms has gained less focus.^[^ ^25^, ^26^, ^27^, ^28^ ^]^ The main advantages of using fibrin gels in 3D cell culture is their inherent ability to promote cell growth and differentiation as well as their tunable mechanical properties.^[^ ^29^ ^]^ For example, by varying the concentrations of fibrinogen and thrombin, stiffness of fibrin gels can be easily adjusted.^[^ ^30^ ^]^ The mechanical microenvironment plays an important role in regulation of cell proliferation, morphology, and differentiation.^[^ ^31^ ^]^ In addition, fibrin represents one of the most strain‐stiffening materials among biopolymer gels.^[^ ^32^ ^]^ Stiffening upon increasing strain has been observed in hydrogels derived from certain cytoskeletal or ECM components and polysaccharides but less commonly in synthetic gels.^[^ ^33^, ^34^, ^35^ ^]^ For fibrin, strain‐stiffening is expected to be an important feature for maintaining blood clot integrity in vivo and may be involved in long range cell‐cell communication in vitro.^[^ ^36^, ^37^ ^]^

In this work, the aim is to explore phenotypic plasticity of breast cancer cells in vitro using 3D fibrin gels. As a model for triple negative CTCs, we use DU4475 cells that were established from a cutaneous metastatic nodule of an advanced breast cancer and grown in free‐floating conditions.^[^ ^38^ ^]^ Culturing DU4475 cells in fibrin gels of two different stiffnesses leads to distinct cell aggregate morphologies, demonstrating the importance of mechanoregulation for DU4475 cell phenotype. Moreover, cell morphology, proliferation and gene expression in fibrin gels clearly differ from BME‐based and suspension cultures. Considering the apparent role of fibrin in cancer, the developed scaffolds provide a potential platform to uncover cancer cell adaptive mechanisms to matrix‐induced cues encountered by CTCs during metastasis.

## 2 Results

### 2.1 Physicochemical Optimization of Fibrin Gels for 3D Breast Cancer Cell Culture

**Figure 1:**
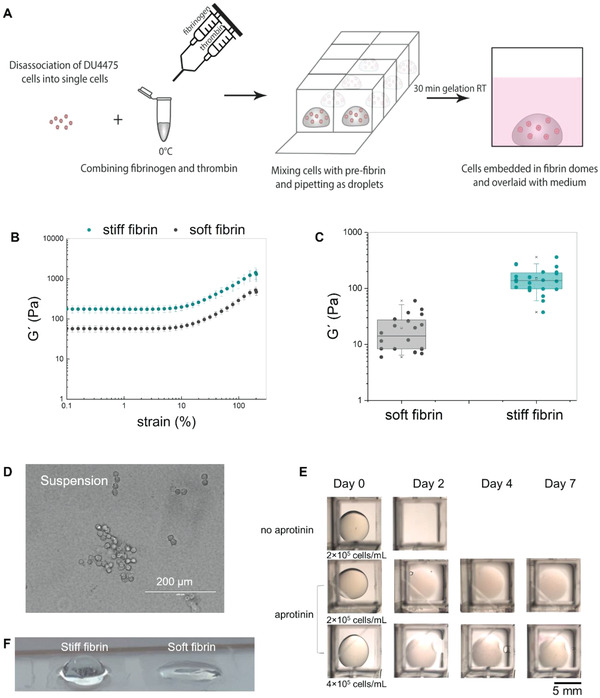
Fibrin gels for 3D cell culture of DU4475 breast cancer cells. A) Schematic of cell encapsulation in 3D fibrin gels. B) Strain‐stiffening properties of fibrin gels measured in bulk rheometer. Storage modulus *G′* represents elastic properties of the gel. Data are presented as the average with standard deviation as error bars (*n* = 3). C) Box plot of *G′* values measured in microrheometer. The data points from each sample are arranged into columns to visualize intrasample heterogeneity. Location of 3–6 microparticles was tracked in each sample (*n* = 4–5). D) Light microscopy image of DU4475 cells grown in suspension. E) Soft fibrin gels seeded with DU4475 cells were stable when 100 U mL^−1^ aprotinin was added to the culture medium. Seeding density of 2–4 × 10^5^ cells mL^−1^ was tested (*n* = 3). F) Shape of fibrin gel droplets after 7 days in culture.

Fibrin gels were prepared utilizing an enzymatic reaction between fibrinogen and thrombin using a modified literature procedure.^[^ ^39^ ^]^ Prior to enzymatic polymerization the reagents were cooled to ≈0 °C to allow easy handling of the pre‐gel solution. The gelation kinetics of fibrin was sufficiently slowed down at low temperature, allowing to embed cells inside fibrin gels before gelation was complete (**Figure** **1**A). As the exact mechanical composition of native fibrin clots that CTCs interact with in vivo remains unclear, we used two different concentrations of fibrinogen to obtain fibrin gels of varying stiffness. Hereafter, we refer to these gels as soft fibrin (10 mg mL^−1^) and stiff fibrin (30 mg mL^−1^). The mechanical properties of the gels were characterized using rheology, which is a suitable technique for measuring bulk properties of soft viscoelastic materials. Here, stiffness was defined as the ability of the gels to resist deformation under oscillatory shear, represented by the shear storage modulus (*G′*). At both fibrin concentrations, *G′* was over a magnitude higher than shear loss modulus (*G″*), indicating that fibrin gels exhibited viscoelastic solid‐like properties (Table S1, Supporting Information). The average *G′* was 57 ± 8 Pa in soft fibrin and 175 ± 29 Pa in stiff fibrin. The values of *G′* corresponded to Young's modulus (*E*) of 171 Pa and 525 Pa in soft fibrin and stiff fibrin, respectively. These results suggest a roughly linear relationship between fibrinogen concentration and gel stiffness in these formulations. Although the initial stiffness of our fibrin gels can be considered rather soft (*G′*< 1 kPa), both concentrations showed remarkable strain‐stiffening, starting ≈10% strain and resulting in a tenfold increase in *G′* at 100% strain (Figure 1B).

In addition to bulk rheology, we used magnetic microrheology to study spatial microscale mechanical heterogeneity of fibrin gels (Figure S1, Supporting Information).^[^ ^40^ ^]^ The method used herein was based on embedding cell‐size‐scale magnetic microparticles within the gel and following their nanometer‐length‐scale movement along a magnetic‐field gradient. The used particle size (≈30 µm) was considerably larger than the fibrin mesh size (Figure S2, Supporting Information) and thus, the particle movement was determined by the local fiber network and its mechanical properties. While the microrheology showed lower average stiffness for soft fibrin than the bulk rheology (i.e., mean values of 20 Pa in microrheometry versus 57 Pa in parallel plate rheometry) (Figure 1B,C), the observation is not surprising as the measured mechanical properties of soft materials can vary by several folds from one method to another.^[^ ^41^, ^42^, ^43^ ^]^ Therefore, we evaluated intrasample heterogeneity based on the distribution of *G′* values rather than by comparing absolute *G′* values. For this, we calculated the coefficient of variation (CV, standard deviation divided by average value) of *G′* values in each sample. The CV ranged from 42% to 106% in soft fibrin and 25% to 58% in stiff fibrin (Figure S3, Supporting Information). Such high CV values suggest the presence of mechanical heterogeneity within gels at both fibrin concentrations. The variability of the CV also implies that there has been slight variation in the micromechanical properties between replicate samples. Regardless, there was practically no overlap of *G′* values between soft and stiff fibrin gels (Figure 1C). This suggests that the two fibrin concentrations provide clearly distinct mechanical microenvironments for cells.

For CTC model we selected the DU4475 cell line, which originates from a metastatic lesion. DU4475 cells represent TNBC subtype and have features resembling CTC biology. Like CTCs, these cells grow in suspension and express epithelial markers, such as cytokeratin 8 (CK8) and epithelial cell adhesion molecule (EpCAM).^[^ ^44^, ^45^, ^46^ ^]^ DU4475 cells form cord‐like clusters in suspension culture (Figure 1D), therefore, the cells were trypsinized before seeding. Single cells were then encapsulated in fibrin gel droplets and cultured for 7 days. The addition of fibrinolysis inhibitor aprotinin into the cell culture medium was necessary to avoid the degradation of fibrin gels during cell culture (Figure 1E). Addition of aprotinin led to remarkable fibrin gel stability and retained gel diameter constant throughout the culture period. The stability was maintained even when cell seeding concentration was increased from 2 × 10^5^ to 4 × 10^5^ cells mL^−1^, indicating that fibrinolysis was efficiently prevented. We also observed that stiff fibrin gels maintained a dome‐shape, whereas soft fibrin gels became more flattened out during culture (Figure 1F). This is likely due to the faster gelation of stiff fibrin, allowing the dispensed droplet to be constricted to its initial shape. In addition, both fibrin gels were optically transparent and thus allowed microscopical examination of the cells.

### 2.2 Soft Fibrin Gels Promote DU4475 Proliferation While Stiff Fibrin Gels Induce Cellular Protrusions

**Figure 2:**
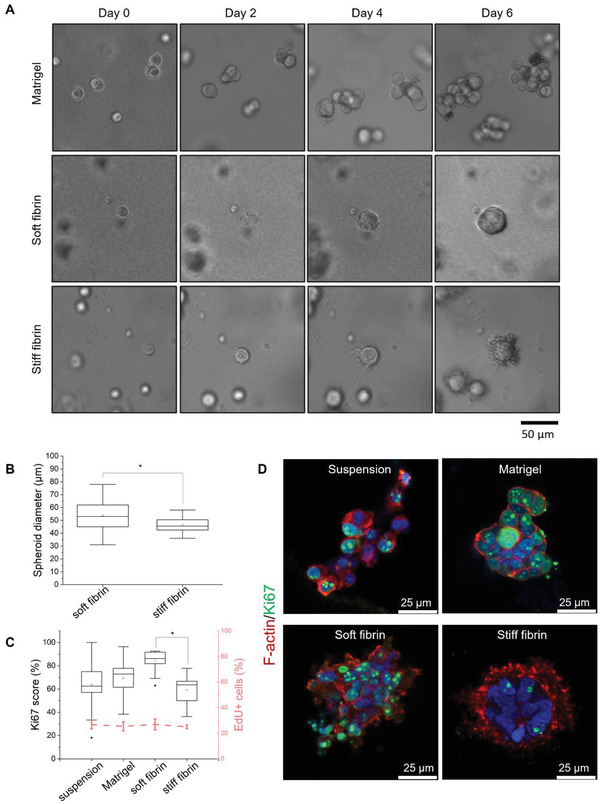
Phenotype and proliferation of DU4475 cells cultured in different matrices for 7 days. A) Light microscopy images of cell growth in different matrices over time. B) Box plot of spheroid diameter (*n* ≥ 15 spheroids). C) Proliferation rate was estimated using the Ki67 score (box plot, white) and EdU incorporation (line graph, pink). Ki67 score shown in the box plot was obtained by dividing the number of Ki67‐expressing cells by the total number of counted cells from two independent cultures (*n* ≥ 200 cells). The fraction of EdU‐positive cells was obtained by counting cells from three independent cultures (*n* ≥ 600 cells). The error bar represents the standard deviation. The asterisks denote cultures with significant differences in ANOVA with Tukey's HSD post‐hoc analysis (*p* < 0.05). D) Representative confocal images of cells showing Ki67 (green), F‐actin 8 (red), and nuclei (blue) staining.

To investigate the effect of mechanical properties of fibrin gels on DU4475 cell phenotype, we monitored changes in cell growth and morphology over time (**Figure** **2**A). DU4475 cells grown in suspension culture served as the control for original cell phenotype. For comparison, cells were also cultured in growth factor reduced Matrigel, a commercial BME composed of laminin, collagen IV and other basement membrane components extracted from mouse Engelbreth‐Holm‐Swarm sarcoma.^[^ ^47^ ^]^ Matrigel has been reported to have G′ values in the range of 50–90 Pa, which is similar to the stiffness of the soft fibrin gel used in this work.^[^ ^48^, ^49^ ^]^

All three matrices promoted cell growth and assembly into multicellular clusters. However, the cluster morphology was markedly different. In Matrigel, the cells formed loosely packed aggregates of varying shapes and sizes. On the contrary, fibrin gels promoted the formation of compact spheroids. The spheroid diameter in soft fibrin ranged from 30 to 80 µm, with an average diameter of 54 ± 11 µm (Figure 2B). Spheroids in stiff fibrin were slightly smaller with an average of 47 ± 6 µm. Notably, the cells in stiff fibrin gels developed protrusions directed toward the matrix. The protrusions first appeared ≈day 2–4. The length and abundance of the protrusions increased during the rest of the culture period. We also observed that the protrusions stained positive for F‐actin, indicating that they were part of the cytoskeletal network (Figure 2C).

As spheroid growth and cell morphology are closely linked to proliferation,^[^ ^50^, ^51^ ^]^ we assessed the expression of proliferation markers using immunofluorescent confocal microscopy. The proliferation rate was estimated using 5‐ethynyl‐2′‐deoxyuridine (EdU) staining, a thymidine analog incorporated into newly synthesized DNA. The percentage of EdU‐positive cells was comparable in suspension and in matrix cultures (Figure 2C). Additionally, we determined the percentage of cells positive for proliferation marker Ki67, which is a protein present throughout all stages of the cell cycle except during quiescence. The percentage of Ki67‐positive cells (Ki67 score) was high in all conditions, with mean values above 60% (Figure 2C,D). The highest Ki67 score was observed in cells cultured in soft fibrin, and the difference was significant compared with the Ki67 score in stiff fibrin. Thus, while cells in all gels exhibited similar DNA synthesis rates, the higher proportion of Ki67‐positive cells in soft fibrin indicates that the cells were in a more actively dividing state than those in stiff fibrin.

### 2.3 Suspension and Matrix‐Based Culture Conditions Establish Specific Gene Expression Programs in DU4475 Cells

**Figure 3:**
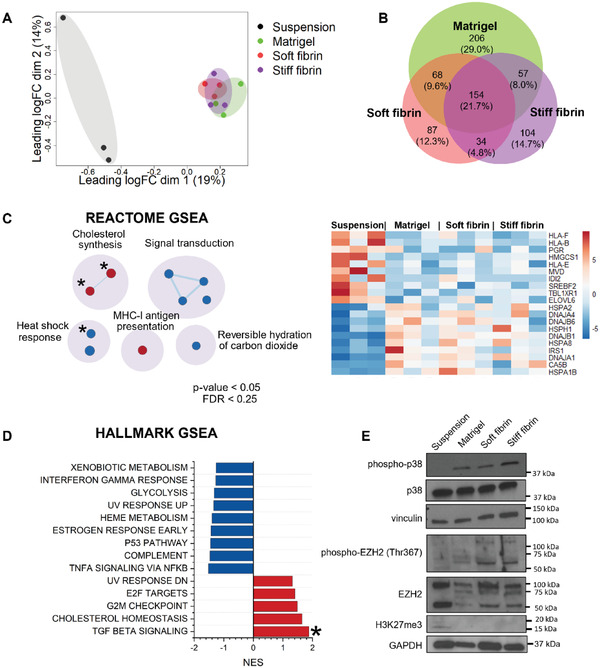
Gene expression patterns of matrix and suspension DU4475 cell cultures. A) Principal component analysis (PCA) of gene expression. B) Venn diagram of differentially expressed genes between cells cultured in matrices and suspension. C) Enrichment map of Reactome processes enriched in suspension culture compared to cells grown in matrices. The heatmap shows row normalized counts of core up and downregulated genes in enriched Reactome processes. D) Bar plot of Hallmark processes enriched in suspension culture compared to cells grown in matrices. E) Representative image of a Western blot probed for p38 and EZH2 activity (*n* = 3). Phospho‐p38 represents the active form of p38, phospho‐EZH2 (Thr367) is the inactive form of EZH2, and H3K27me3 is the histone H3 trimethylated at lysine 27 by active EZH2. Vinculin and GAPDH serve as loading controls. For enrichment results, processes with FDR < 0.25 and *p* < 0.05 in GSEA are shown. The asterisks denote gene sets with FDR < 0.05 and *p*‐value < 0.001. RNA‐sequencing analysis was done on samples from three independent cultures. FDR = false discovery rate.

To explore the matrix‐dependent effects on gene expression programs in DU4475 cells, we performed total RNA sequencing analysis for the cells grown in different culture conditions. Principal component analysis (PCA) and Venn diagram analysis demonstrated that matrix embedment of the cells leads to global reprogramming of gene expression (**Figure** **3**A,B). In PCA, the matrix‐cultured cells clearly clustered separately from the suspension cells (Figure 3A). Furthermore, almost 22% of differentially expressed genes (DEGs) between matrix‐culture and suspension culture were shared between all matrices (Figure 3B). PCA also showed that there is more variation within suspension replicates than between matrices, which difference likely arises from varying growth phase of suspension cells at cell harvest (Figure 3A). The reason is that in suspension cultures, the cell density of multicellular clusters could not be accurately estimated, whereas in matrix‐cultures equal number of single cells was seeded into each gel.

Instead of focusing on gene expression at single gene level, we performed gene set enrichment analysis (GSEA) to reveal changes in biological processes.^[^ ^52^ ^]^ For the initial screening, we compared suspension cells and cells grown in matrices using established collections from the Molecular Signatures Database (MSigDB). The main enriched gene sets in the Reactome collection were involved in cholesterol synthesis and major histocompatibility complex‐I (MHC‐I) mediated antigen presentation (Figure 3C). Cholesterol synthesis is associated with TNBC metastasis, whereas antigen presentation by MHC‐I is characteristic for DU4475 cells and immunomodulatory subtype of TNBC.^[^ ^53^, ^54^, ^55^ ^]^ In contrast, matrix‐cultured cells were enriched in signal transduction pathways, heat shock response and hydration of carbon dioxide, which are processes promoting cell recovery in stressful conditions.^[^ ^56^, ^57^ ^]^ Likewise, similar processes were identified using the Hallmarks collection (Figure 3D). Whereas suspension cells were most enriched in Hallmarks of cell cycle regulation, matrix‐cultured cells were enriched in Hallmarks involved in stress responses, such as signaling by NF‐κB and p53 (Figure 3D).^[^ ^58^, ^59^ ^]^

To further validate the presence of stress signaling in matrix‐cultured cells, we examined the activation of stress response protein p38 using Western blot analysis. It has been previously demonstrated that upregulation of p38 signaling in patient‐derived breast epithelial and breast cancer explant cultures depends on matrix stiffness.^[^ ^60^ ^]^ Similar trend was observed with DU4475 cells, as the expression of activated p38 (phosphorylated p38) was upregulated in cells cultured in matrices and was very low or absent in suspension cells (Figure 3E). Compressive stress and activated p38 have also been shown to downregulate expression of Enhancer of zeste homolog 2 (EZH2), which is the catalytic subunit of an epigenetic regulator polycomb repressive complex 2 (PCR2).^[^ ^60^, ^61^ ^]^ Accordingly, expression of total EZH2 and its target trimethylated lysine 27 on histone H3 (H3K27me3) decreased in matrix‐cultured cells compared to suspension cells (Figure 3E). Furthermore, the amount of EZH2 inactive form (phosphorylated at threonine 367) increased in fibrin gels. However, there were no significant differences in phosphorylated p38 or EZH2 expression between soft and stiff fibrin, most probably due to all matrices being in a relatively soft stiffness range (*G′* < 1 kPa).

### 2.4 DU4475 Cell State in Matrix‐Based Cultures Corresponds to Matrix Composition and Stiffness

**Figure 4:**
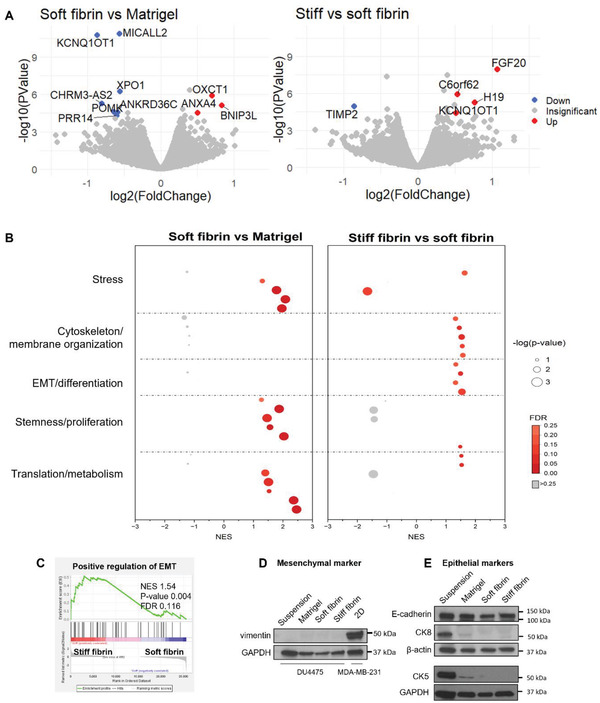
Matrix stiffness‐ and composition‐specific impacts on the phenotype of DU4475 cells. A) Volcano plot of differentially expressed genes between soft fibrin and Matrigel, and stiff fibrin and soft fibrin. Gene names with FDR < 0.05 and |logFoldChange| > 0.5 are shown. Red represents upregulated and blue represents downregulated genes and grey nondifferentially expressed genes B) Bubble plot of gene sets enriched in soft fibrin versus Matrigel and in stiff fibrin versus soft fibrin. The size of the bubbles corresponds to ‐log(*p*‐value) and the color to the FDR. C) The enrichment plot for positive regulation of EMT in stiff fibrin is shown. D) Representative Western blots for mesenchymal marker vimentin and E) epithelial markers (E‐cadherin, CK8, CK5) are shown. GAPDH and β‐actin serve as loading controls. MDA‐MB‐231 cells serve as positive control for vimentin expression. NES = normalized enrichment score, FDR = false discovery rate. RNA‐sequencing analysis was done on samples from three independent cultures.

Next, we analyzed matrix‐specific effects on gene expression by comparing matrices of similar stiffness but different protein composition and network structure (Figure S2, Supporting Information) (soft fibrin vs Matrigel), and matrices of similar composition but different stiffness (stiff vs soft fibrin). Only a handful of DEGs were observed in these comparisons, indicating that despite contrasting cell morphology, gene expression differences were subtle (**Figure** **4**A). As the most upregulated DEG in stiff fibrin was FGF20, we used infigratinib to inhibit fibroblast growth factor receptor (FGFR) signaling. However, this had no effect on protrusion formation (Figure S4, Supporting Information).

For more targeted GSEA of matrix‐induced changes, we created a panel with gene sets considered relevant for the observed phenotypes, such as those associated with differentiation, cytoskeleton organization, and metabolism regulation (Table S2, Supporting Information**)**. Several differences were observed when comparing soft fibrin and Matrigel (Figure 4B). Compared to Matrigel, cells in soft fibrin showed strongly enriched gene sets of translation and metabolism, as well as in processes associated with stemness and progenitor cell regulation. In addition, stress responses were enriched in soft fibrin. The differences between fibrin gels of varying stiffness were smaller (Figure 4B), as evidenced by lower ‐log(*p*‐values). Nonetheless, cells in stiff fibrin showed enrichment of gene sets corresponding to regulation of cell differentiation. As expected from the protrusion formation, gene sets in cytoskeleton regulation were also enriched in stiff fibrin compared to soft fibrin. In addition, gene set for positive regulation of epithelial‐mesenchymal transition (EMT) was enriched (Figure 4D), suggesting that cells in stiff fibrin were switching toward a more mesenchymal‐like phenotype compared to soft fibrin. However, conventional markers for EMT (loss of E‐cadherin and gain of vimentin) did not show up on protein level (Figure 4E,F), indicating that the cells retained their epithelial characteristics. Conversely, epithelial cytokeratins CK8 and CK5 decreased in all matrices compared to suspension (Figure 4E). Together, the data suggest that a transition in cell state was occurring in all matrices but did not lead to complete EMT.

### 2.5 Stiff Fibrin Gel Induced Cell Protrusions Arise From Actin‐ and Tubulin‐Rich Blebs

**Figure 5:**
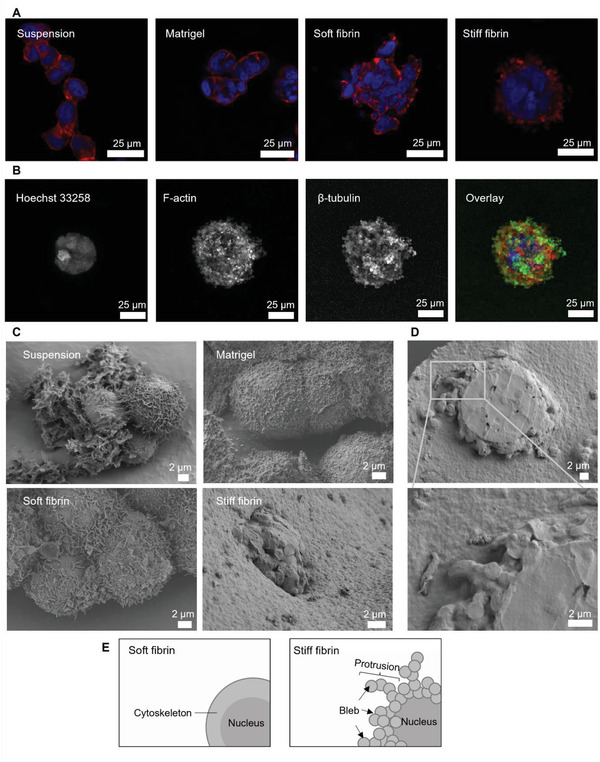
Confocal immunofluorescence and electron microscope analysis of protrusions. A) Organization of nuclei (blue) and F‐actin (red) differs in DU4475 cells grown in different conditions. B) Maximum intensity projection revealed the protrusions formed in stiff fibrin are composed of bleb‐like structures that contained F‐actin (red) and β‐tubulin (green). C) Cells grown in suspension, Matrigel and soft fibrin displayed microvilli, whereas spheroids formed in stiff fibrin had bleb‐like surface. D) Protrusive spheroids were cross‐sectioned to better observe cell organization within the spheroid. Magnification shows rounded morphology of individual protrusions. E) Schematic representation of cell morphology and protrusion structure in soft and stiff fibrin.

Both phenotype conversion and protrusion formation are associated with invasive processes.^[^ ^62^ ^]^ Therefore, we set out to characterize the protrusions formed in stiff fibrin to elucidate their role for the cells. First, we investigated how cytoskeletal organization differed between cells grown in different matrices by comparing spheroid cross‐sections. We observed that in stiff fibrin, the area occupied by F‐actin was on average 43% of spheroid area. F‐actin coverage was 37% and 21% in soft fibrin and Matrigel, respectively (**Figure** **5**A). In soft fibrin and Matrigel, F‐actin appeared to be evenly distributed around the cells. On the other hand, in stiff fibrin F‐actin of cells was concentrated at the spheroid perimeter in the protrusions, displaying a branched structure with rounded, bleb‐like ends (Figure 5A,E). These protrusions had a diameter between 1.5 and 4.0 µm. Although the protrusions were symmetrically distributed around each spheroid, their lengths varied between 2 and 14 µm in different spheroids. We also observed β‐tubulin at the periphery of the protrusions (Figure 5B), which has been reported to localize into blebs.^[^ ^63^, ^64^ ^]^

Additionally, we investigated the dynamics of protrusion formation. Although long protrusions were established over several days, live cell imaging revealed that the structures were highly motile (Movie S1, Supporting Information). Protrusions actively extended and retracted at their tips, resulting in net protrusion growth during culture. The lifetime of individual protrusions was in the time scale of minutes to hours, which is longer than the dynamics of bleb expansion and retraction that are considered to occur very rapidly.^[^ ^65^ ^]^

To observe protrusion structure at higher resolution, we performed scanning electron microscopy (SEM) analysis of the spheroids (Figure 5C). DU4475 cells in suspension, Matrigel and soft fibrin formed rather loose structures that displayed microvilli on cell surface. In stiff fibrin, numerous small blebs were observed on spheroid surface. Cross sections of these spheroids showed clustered nuclei surrounded by a capsule of bleb‐like protrusions (Figure 5D,E). These observations correlate well with the protrusion structure observed in confocal microscopy. In addition, SEM analysis showed that the spheroids in fibrin gels were surrounded by a dense matrix. This indicates that the presence of aprotinin effectively inhibited fibrin degradation and cells remained mechanically supported throughout the culture period.

### 2.6 Protrusion Formation is Mechanically and Spatially Regulated

**Figure 6:**
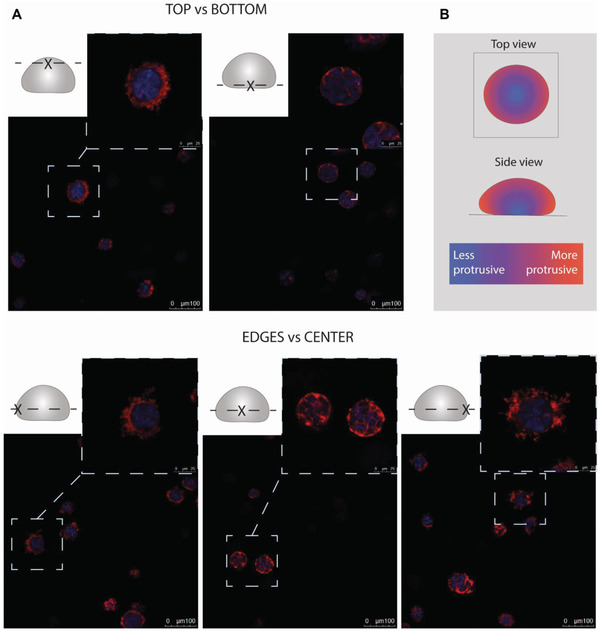
Protrusion formation depends on cell position within stiff fibrin gel. A) Protrusive DU4475 spheroids were enriched at top and edges of the gel. Inset shows the location where the image was taken. The cells were stained for nuclei (blue) and F‐actin (red). Scale bar is 100 µm in the broad view and 25 µm in the close‐up. B) Schematic of protrusive cell enrichment in different parts of the gel.

The protrusions only formed in stiff but not soft fibrin, suggesting a mechanobiological mode of cell regulation. To study the hypothesis further, we explored whether mechanical and spatial cues from the matrix could dynamically, reversibly, and locally promote the genesis of protrusions. First, we investigated cues leading to protrusion formation. Surprisingly, we noticed that protrusive cells were not equally distributed between different parts of the stiff fibrin gel (**Figure** **6**A). The spheroids with protrusions were clearly enriched at the edges and top parts of the gel, with more round spheroids localizing to the bottom and the middle of the gel (Figure 6B). This brings up the intriguing possibility that the position within the stiff fibrin gel influences the local mechanical environment of the cells, although we were not able to confirm this with the current micro rheological setup. To exclude that the stiff substrate was influencing the mechanical environment perceived by the cells, we detached fibrin gel droplet from the bottom of cell culture dish after polymerization to perform so called floating culture. The method is used to study the development of mechanical tension as floating culture allows cells to contract the gel.^[^ ^66^ ^]^ However, floating gel culture resulted in similar protrusion formation as in anchored culture (Figure S5, Supporting Information). To further investigate the role of contraction in protrusion regulation, we used RhoA inhibitors (Rhosin and CT04) and calmodulin antagonist (W7) to interfere with myosin light chain ‐mediated actomyosin contractility. The results suggest that the protrusions were governed by a mechanism independent of RhoA signaling (Figure S6, Supporting Information). However, the involvement of calmodulin in mechanical transduction could not be determined due to cell death after treatment with W7 (Figure S6, Supporting Information).

**Figure 7:**
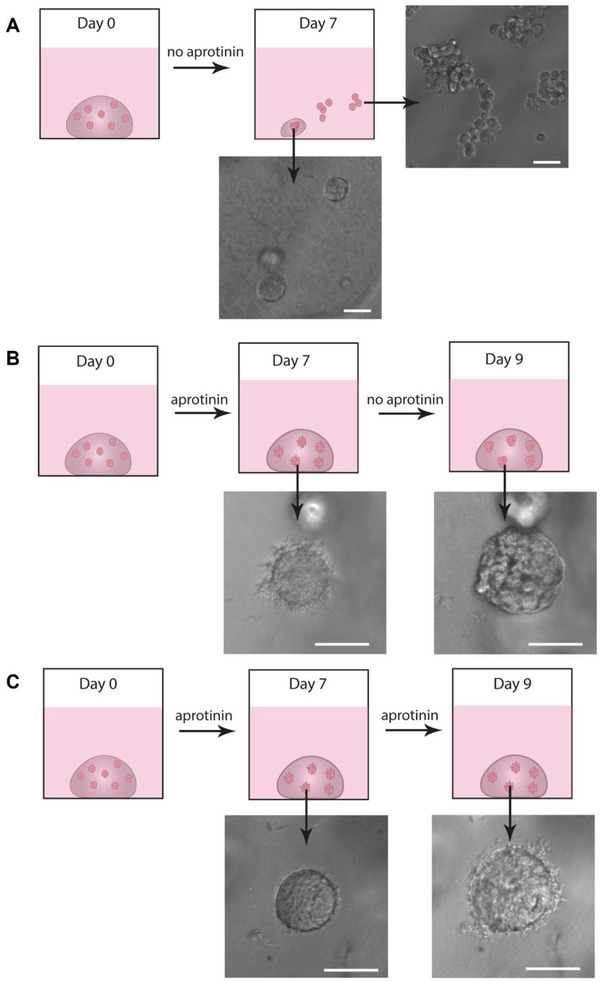
The fibrin stiffness dynamically regulates genesis of the DU4475 protrusions. A) Stiff fibrin gels degraded almost completely in the absence of aprotinin. After 7 days, most DU4475 cells were freely floating in cell culture medium and some non‐protrusive spheroids remained embedded in soft gel slices. B) Cessation of aprotinin supplementation on day 7 led to loss of protrusions. C) When aprotinin was supplied throughout the culture period, protrusion growth persisted. Scale bar is 50 µm.

To study the relationship between fibrin mechanical properties and protrusions further, we investigated how fibrin degradation and subsequent softening of the matrix affects protrusions. Two complementary setups were used to address this question (**Figure** **7**): 1) no aprotinin was used, or 2) aprotinin supplementation was ceased at day 7. When no aprotinin was added to the culture (Figure 7A), stiff fibrin gels were almost completely degraded at the end of the 7‐day culture period. Thereafter, majority of cells were no longer confined in the matrix but floating freely in the cell culture medium. The morphology of these cells resembled the cells grown in suspension culture and the cells lacked the protrusions (Figures 1D and 7A). In places where small fragments of gel were found, cells had assembled into smooth spheroids, resembling the morphology of spheroids grown in soft fibrin (Figures 2A and 7A). Considering the overall degradation of the matrix, stiffness in these gel remnants is expected to be rather low.

In the second setup, cells were grown in stiff fibrin for 7 days, after which the culture was continued for two more days either in the presence or absence of aprotinin (Figure 7B,C). By removing aprotinin only after spheroid formation, more controlled rate of matrix degradation was achieved, and the cells remained inside the gel on day nine. Removal of aprotinin caused spheroids to transition into more relaxed structures and to lose their protrusions (Figure 7B). In case aprotinin supplementation was continued, protrusion growth persisted (Figure 7C). Altogether, the results indicate that sufficient fibrin concentration and stiffness is a driver for protrusion formation and that the cells can switch between protrusive and non‐protrusive phenotypes in response to the local matrix environment in fibrin gels.

## 3 Discussion

The current study addresses how CTC‐like breast cancer cells adapt to versatile matrix cues, a phenomenon which is of relevance to metastasis and the often‐following therapy resistance. In vivo, only a minute fraction of CTCs in circulation are able to survive and form metastases.^[^ ^17^ ^]^ Therefore, identifying the factors promoting CTC survival is the key in the complete prevention of metastasis. One of the main challenges in the CTC research has been the detection and isolation of cells from circulation.^[^ ^67^ ^]^ In this study we utilized a triple negative DU4475 cell line with CTC‐like features as a model for CTCs. Owing to the intriguing link between hypercoagulable state and cancer progression,^[^ ^18^, ^24^, ^68^ ^]^ we prepared 3D fibrin gels of varying stiffness to culture DU4475 cells and to study the interplay between matrix properties and cell response in vitro. To compare phenotypes formed in fibrin gels with a matrix of a different biochemical composition, we used Matrigel, which is BME frequently used to evaluate the metastatic potential of cancer cells.^[^ ^16^, ^47^, ^69^ ^]^ We observed that DU4475 cells responded distinctively to different gels, reflected in differences in cell growth, morphology and signaling pathways.

The changes in DU4475 cell behavior in response to different matrix environments implies the cells show great deal of matrix controlled phenotypic plasticity that is an emerging concept highlighted as a hallmark of cancer.^[^ ^70^ ^]^ Whereas normal cells become differentiated during tissue development, cancer cells are often poorly differentiated and can acquire stem cell ‐like characteristics that enable them to switch phenotypes to better adapt to changes in their microenvironment.^[^ ^70^, ^71^ ^]^ Previous research suggests that the phenotype switching may give rise to drug tolerant cells in TNBC and other cancers.^[^ ^72^, ^73^ ^]^ Phenotypic plasticity is largely mediated through transcriptional control and epigenetic regulation of target genes, including histone modifications and remodeling of chromatin structure.^[^ ^71^ ^]^ Suppression or activation of genes alters the transcriptional profile of the cells, rapidly changing the cell state in response to chemical and physical cues. A central epigenetic regulator is the histone methyltransferase EZH2, whose overexpression correlates with TNBC and poor prognosis.^[^ ^74^, ^75^, ^76^ ^]^ As the catalytic unit of PCR2, EZH2 represses transcription of differentiation associated genes and thus promotes phenotypic plasticity by maintaining cells in a stem cell ‐like state.^[^ ^77^, ^78^ ^]^ Here, we showed that DU4475 cells in suspension expressed high levels of EZH2 as well as co‐expressed basal CK5 and luminal CK8. This indicates that the cells had a phenotype resembling bipotent mammary progenitor cells.^[^ ^77^, ^79^ ^]^ In matrix cultures, cytokeratin expression decreased together with H3K27me3, suggesting transitioning toward cellular differentiation. The shift in cell state is reminiscent of partial (i.e., hybrid, intermediate) EMT, which has been observed in CTCs and is associated with enhanced metastasis formation.^[^ ^80^, ^81^, ^82^, ^83^, ^84^ ^]^

When comparing differences between matrices, we observed that soft fibrin maintained DU4475 cells in a more proliferative progenitor‐like state than Matrigel, as evidenced by high Ki67 score and the enrichment of stemness, translation and metabolism related pathways. In addition, stress‐related gene sets were enriched compared to Matrigel, perhaps due to increased metabolic demand of rapidly dividing cells in soft fibrin. In contrast, cells cultured in stiff fibrin acquired a more differentiated cell morphology and were enriched in gene sets of differentiation. Furthermore, cells in stiff fibrin upregulated FGF20, expression of which is associated with cell differentiation.^[^ ^85^, ^86^ ^]^ FGF20 is one of 22 identified FGFs that regulate cell behavior in a context‐dependent manner. The canonical FGF signaling is mediated through FGF binding to FGFR with heparan sulfate proteoglycan (HPSG) or Klotho as coreceptor.^[^ ^87^ ^]^ However, inhibition of FGFR did not influence cell morphology, either suggesting that FGF20 was not the main cause for phenotype switch, or that FGF20 elicited its biological effects independently of FGFR. This non‐canonical signaling can occur, for example, via the interaction of FGFs with HSPG syndecans.^[^ ^87^ ^]^ Collectively, the results are in agreement with previous reports of fibrin gels in cancer cell culture, where soft fibrin gels have been more successful in maintaining cancer stem cell ‐like features than stiffer ones.^[^ ^88^, ^89^ ^]^ Notably, cells cultured in stiff fibrin were not committed to a differentiated state, as the morphological changes could be reversed by allowing softening of the gel. Such trans‐ or dedifferentiation potential further demonstrates the importance of matrix‐guided regulation of phenotypic plasticity.

The most prominent feature of cells cultured in stiff fibrin was the generation of multiple bleb‐like protrusions toward the matrix. Protrusions are defined as cell membrane projections that represent either pressure‐driven blebs or actin‐driven protrusions, such as filopodia and lamellipodia.^[^ ^90^ ^]^ The classification is typically based on protrusion morphology and differentially expressed actin regulators, although there is overlap between protrusion subtypes.^[^ ^91^, ^92^ ^]^ The classification is further complicated by the fact that most protrusion characterization has been done in 2D, whereas protrusions in 3D might be morphologically and mechanistically different. In 3D, cells can switch between different types of protrusions and this is affected by various factors, such as the degree of adhesion, proteolysis, confinement and matrix stiffness.^[^ ^93^, ^94^, ^95^ ^]^ Actin‐driven protrusions are characteristic of mesenchymal migration on rigid substrates, whereas blebbing is favored when matrix proteolysis is prevented.^[^ ^96^, ^97^ ^]^ Surprisingly, the protrusions in stiff fibrin appeared to have morphological features of both blebs and actin‐driven protrusions. This is reminiscent of “blebbopods” discovered in *Dictyostelium* which is a model organism for cell migration.^[^ ^98^ ^]^ When cultured under stiff agarose overlay, some blebs in these cells develop into pseudopods through continued actin polymerization. Although blebs and actin‐driven protrusions are often described as opposing migration modes, they can also coexist in the same protrusion.^[^ ^94^, ^99^ ^]^ These hybrid protrusion types might represent an under‐characterized cancer cell adaptation mechanisms to mechanical stress. Correspondingly, CTCs have been shown to be morphologically heterogeneous and this is presumably caused by the shear stresses in the bloodstream.^[^ ^100^, ^101^ ^]^

To gain more insights into the genesis of DU4475 cell protrusions, we characterized the regulation of protrusion formation. These investigations revealed an additional level of phenotypic plasticity, as protrusion growth was dependent on culture time and location within fibrin gel, which also mimics the evolution of cancer cell subpopulations observed in vivo.^[^ ^102^, ^103^ ^]^ It is conceivable that the enhanced protrusion formation at the edges of stiff fibrin might be related to the stable dome geometry of the gel, as scaffold shape and curvature is known to guide cell behavior in vitro and in vivo.^[^ ^104^, ^105^, ^106^ ^]^ The diffusion limited transport of gases, nutrients or growth factors into the gel core might promote the observed boundary effect.^[^ ^106^, ^107^, ^108^ ^]^ Importantly, we demonstrated that adequate fibrin stiffness is a pre‐requisite for protrusion formation, implying that features such as curvature‐induced stress or mechanical gradients might have been introduced into the gel dome during polymerization. These forces might subject the cells at edges to higher levels of tension or compression. How the strain‐stiffening behavior or fibrillar structure of fibrin gels contributes to this uneven distribution of stresses is an intriguing question that remains to be clarified.

While protrusions are most often studied in the context of cell migration, we did not observe active migration of DU4475 cells regardless of whether proteolytic degradation of the matrix was inhibited by aprotinin or not. Regardless, protrusions are known to be involved in other important aspects of cancer cell physiology. For example, protrusions may promote invasion by mediating mechanosensing, or by deforming or degrading the ECM.^[^ ^109^, ^110^, ^111^, ^112^ ^]^ A possible explanation is that the protrusions act as a response to counteract compressive stress generated by the surrounding matrix. In the absence of proteolytic degradation, the growing spheroid must displace the surrounding matrix despite the compressive pressure that builds on the cells. A similar phenomenon has been demonstrated in mammary epithelial cell spheroids cultured between polydimethylsiloxane (PDMS) pillars.^[^ ^113^ ^]^ The increase in cellular tension would explain the progressive protrusion growth during culture and the loss of protrusions once fibrinolysis is allowed. The presence of bleb‐like units in the protrusion structure supports the notion of elevated intracellular pressure, as cells may use blebbing as a strategy for pressure release.^[^ ^114^ ^]^ Although Rho signaling is associated with various blebs, its inhibition did not affect protrusion formation in DU4475 cells.^[^ ^96^, ^115^, ^116^ ^]^ The contribution of calmodulin‐dependent blebbing could not be confirmed due to cell death associated with the use of calmodulin antagonists.^[^ ^63^, ^117^ ^]^ Alternatively, it is possible that the function of protrusions resembles those of invadopodia, which are invasive, actin‐based structures associated with proteolytic degradation of the matrix.^[^ ^110^ ^]^ However, proteolysis is not strictly necessary since cells may use nonproteolytic invadopodia to mechanically degrade and invade into the matrix, as has been observed in breast cancer cells cultured in 3D alginate‐Matrigel gels.^[^ ^111^ ^]^

Altogether, the data demonstrate that a versatile array of breast cancer phenotypes can be obtained simply by changing matrix composition or stiffness. The investigation of various hybrid states and their interconversion is expected to shed light on the cellular mechanisms that promote cancer cell survival and approaches to target them. This has significant implications in the case of TNBC which has been proven challenging to treat due to its heterogeneity and lack of therapeutical target molecules such as hormone and growth factor receptors that are present in other major subtypes of breast cancer.^[^ ^55^ ^]^ Recent approaches to address TNBC heterogeneity involve algorithms that stratify TNBC patients according to partial EMT status, opening new possibilities on developing personalized treatments.^[^ ^118^ ^]^ However, more comprehensive understanding of how the phenotypic plasticity is regulated by ECM cues is needed. As a natural protein in the human body, fibrin gels are ethical and physiologically relevant alternatives to widely used Matrigel, particularly considering fibrin(ogen)’s implications in promoting CTC survival. Further research is necessary to gain more insights into the in vivo significance of observed DU4475 cell phenotypes and to determine whether the protrusions might be involved in metastasis‐promoting phenomena. Additionally, single cell characterization is expected to capture phenotypic heterogeneities within the matrix more accurately than bulk RNA analysis. With advances in CTC technologies, the developed fibrin gel compositions could be combined with patient‐derived CTCs in the future.

## 4 Conclusions

Due to fibrin's nuanced relevance for cancer progression, we proposed the use of fibrin gels together with CTC‐like cells to study phenotypic plasticity of breast cancer cells. We show that unlike suspension or BME‐based 3D cultures, fibrin gels induced compact spheroid formation of DU4475 cells. Furthermore, we observed the appearance of actin and tubulin containing cellular protrusions in stiff fibrin gel. GSEA and protein expression studies coupled the cell phenotypic alterations to specific expression patterns, with association with partial epithelial‐mesenchymal transition. Although the in vivo significance of the matrix‐driven protrusions warrants further investigation, the study demonstrates how the biological state of cancer cells can be regulated by adjusting the mechanical properties of fibrin gels. Therefore, the developed fibrin scaffolds offer a facile platform to study cancer cell adaptation mechanisms in response to cues from the intravascular ECM. Modeling these interactions in fibrin gels may be of great importance for unveiling the factors that promote CTC survival and metastatic spreading of TNBC.

## 5 Experimental Section

### Cell Culture

DU4475 cells were obtained from the American Type Culture Collection (ATCC). The cells were tested mycoplasma‐free and grown in suspension culture in RPMI 1640 medium (Thermo Fisher Scientific) supplemented with 10% fetal bovine serum (FBS) (Biowest), 2 mm L‐glutamine (Thermo Fisher Scientific) and 1% penicillin‐streptomycin (Thermo Fisher Scientific). The cells were maintained in a humidified incubator at 37 °C and 5% CO_2_ atmosphere.

### Preparation of Fibrin Gels

Lyophilized fibrinogen (F3879, Sigma‐Aldrich) and thrombin (T6884, Sigma‐Aldrich) from human plasma were used to prepare gels with 10 or 30 mg mL^−1^ fibrin. For each 2.5 mg of fibrinogen, 1 NIH U thrombin was used. First, stock solutions were prepared by dissolving fibrinogen in PBS‐Tris buffer (0.5× PBS, 50 mm Tris, pH 8.8) and thrombin in 0.1% bovine serum albumin (BSA) (Biowest). Required volumes of fibrinogen and thrombin stock solutions were diluted into PBS‐Tris buffer and Milli‐Q water, respectively. Prior to use, all solutions were chilled in a cooling block. The solutions were taken in equal volumes into a dual barrel syringe (modified from Viscous Delivery System, Arthrex) and extruded through the mixing tip to initiate the reaction. The pre‐gel was maintained in a cooling block until use.

### 3D Cell Culture

Before encapsulation into 3D gels, DU4475 cells were processed into a single cell suspension. Cells were trypsinized using 0.05% trypsin‐EDTA (Thermo Fisher Scientific) for 10 min at 37 °C and resuspended in PBS. Cells were applied to Nunc Lab‐Tek 8‐well chamber slides (Thermo Fisher Scientific) as 2 µL droplets and mixed with fibrin pre‐gel. For each cell droplet, 40 µL of fibrin gel was used. For the cultures intended for immunofluorescence staining or scanning electron microscopy, the final seeding density in the gel was ≈2 × 10^5^ cells mL^−1^ to allow clear visualization of the cells. The seeding density for the cultures intended for Western blot analysis was twice as high to ensure sufficient protein production. For comparison, cells were seeded in 10 mg mL^−1^ growth factor reduced Matrigel (356 230, Corning). Gelation was allowed to occur for 30 min at room temperature, after which 500 µL RPMI medium with 100 U mL^−1^ aprotinin (10 820, Sigma‐Aldrich) was added on top of fibrin gels and plain RPMI medium on top of Matrigel. Cells were cultured in the gels for 7 days and the medium was changed every 2–3 days. Cell growth was followed with EVOS FL inverted microscope (Thermo Fisher Scientific).

### Fibrin Gel Stability

To ensure the stability of fibrin gels throughout the 7‐day culture period, effect of aprotinin (10 820, Sigma‐Aldrich) addition was tested. 10 mg mL^−1^ fibrin gel was used for the study since the lowest concentration was assumed to degrade at the fastest rate. The gels were prepared at seeding densities of 2 × 10^5^ or 4 × 10^5^ cells mL^−1^ and imaged with a fluorescence stereo microscope Leica MZ FLIII with PLAN APO 1.0× objective (Leica Microsystems) connected to a AxioCam MRc (ZEISS). The gels were then maintained in RPMI medium with or without 100 U mL^−1^ aprotinin in standard cell culture conditions at 37 °C. Change in diameter was followed every 2–3 days accompanied by medium exchange.

### Immunofluorescence Staining

At the end of the culture period, 3D cultures were briefly washed with PBS. For the control, cells from suspension culture were embedded in 10 mg mL^−1^ fibrin and directly processed for immunostaining. Cells in fibrin gels were fixed with 4% paraformaldehyde in PBS for 15 min at room temperature, while cells in Matrigel were fixed only 5 min to avoid depolymerization of the matrix. Gels were washed with PBS 3 × 5 min, after which cells were permeabilized with 0.25% Triton X‐100 in PBS for 20 min at room temperature and washed 3 × 10 min with immunofluorescence (IF) buffer (7.7 mm NaN_3_, 0.1% BSA, 0.2% Triton, 0.05% Tween20 in PBS). Non‐specific binding sites were blocked by incubating the gels 1 h at room temperature with 10% normal goat serum (Thermo Fisher Scientific) in IF buffer. Cells were incubated with primary antibody in blocking buffer for 24 h at 4 °C. Cells were then washed 3 × 20 min with IF buffer and incubated with the appropriate secondary antibody in blocking buffer overnight at 4 °C. To counterstain nuclei and stain F‐actin, cells were washed with IF buffer as previously and incubated 10–20 min with Hoechst 33 258 dye (B2883, 1:10 000, Sigma‐Aldrich) and AlexaFluor 546 ‐conjugated phalloidin (A22283, Thermo Fisher Scientific), after which the gels were washed 2 × 5 min with PBS. Immunostained samples were mounted with Shandon Immu‐Mount mounting medium (Thermo Scientific) and imaged with a confocal laser scanning microscope Leica TCS SP8 MP CARS with a HC PL APO 20×/0.75 or 40×/1.10 water CS2 objective (Leica Microsystems). Primary antibodies were used against Ki67 (ab15580, Abcam, 1:100), cleaved caspase 3 (9661, Cell Signaling Technology, 1:400) and β‐tubulin (ab6046, Abcam 1:200). Secondary antibody was either AlexaFluor 546 ‐conjugated goat anti‐mouse IgG antibody (A‐11003, Thermo Fisher Scientific, 1:300) or AlexaFluor 488 ‐conjugated goat anti‐rabbit IgG antibody (A‐11008, Thermo Fisher Scientific, 1:300).

### EdU Assay

EdU Staining Proliferation Kit (iFluor 488) (ab219801, Abcam) was used to evaluate the proliferation rate of cells grown in 3D cultures for 7 days. The cells were incubated with 10 µm EdU for 4 h under standard cell culture conditions, after which the EdU reaction was carried out according to manufacturer's instructions. Hoechst 33 258 staining and confocal imaging were performed as described in the previous section. The ratio of EdU positive cells/total cells was obtained by counting at least 600 nuclei in total from three independent cultures.

### Inhibitor Studies

1–10 µm FGFR inhibitor (BGJ398, Selleckhem), 25–50 µm calmodulin inhibitor (W7, Medchemexpress), 2 µg mL^−1^ Rho inhibitor (CT04, Cytoskeleton), or 30–100 µm RhoA inhibitor (Rhosin, Selleckhem) were added on day 0 or day 5 to cell culture medium of cells in 30 mg mL^−1^ fibrin. Cell death was assessed by incubating the cells with 1:2000 CellTox green (Promega) for 30 min before fixation, or by immunostaining cleaved caspase 3.

### Western Blotting

3D cultures and control sample from suspension culture were briefly washed with cold PBS. To lyse the cells, samples were incubated with radioimmunoprecipitation assay buffer (150 mm NaCl, 1% Triton‐X, 0.5% sodium deoxycholate, 0.1% SDS, 50 mm Tris, pH 8.0) supplemented with protease and phosphatase inhibitors (Roche) on ice for at least 10 min. Cell nuclei in control sample and Matrigel were broken by extruding the samples several times through a 25 G needle. Cells in fibrin gels were released and lysed by homogenizing the gels for 2 × 15 s at 5000 rpm in Precellys 24 Tissue Homogenizer using lysing kit with CK28 beads (Bertin Technologies). All samples were then centrifuged for 15 min at 16 100 g at 4 °C to pellet the cell debris. Protein concentration of the lysates was determined with *DC* protein assay kit (Bio‐Rad) according to the manufacturer's instructions. ≈7–20 µg of lysate protein was mixed with 5× Laemmli sample buffer (400 mm Tris, 10% SDS, 6% glycerol, 20% β‐mercaptoethanol, 0.25% bromophenol blue, pH 6.8) and incubated 5 min at 95 °C to denature the proteins. Proteins were separated on 4%–20% Mini‐PROTEAN TGX Precast Protein Gels (Bio‐Rad) and blotted onto a 0.2 µm nitrocellulose membrane with Trans‐Blot Turbo Transfer pack (Bio‐Rad). The membrane was blocked with 2%–5% BSA or 5% skim milk in TBST (TBS, 0.05% Tween20) for 45 min at room temperature and incubated with primary antibody in blocking buffer overnight at 4 °C. The membrane was then washed with TBST. Appropriate horseradish peroxidase ‐conjugated secondary antibody in blocking buffer was incubated with the membrane for 1 h at room temperature, after which the washes were repeated. Protein bands where then detected by the enhanced chemiluminescence method using SuperSignal West Femto kit (Fisher Scientific). Primary antibodies were used against phospho‐p38 MAPK (4511S, Cell Signaling Technology, 1:1000), p38 MAPK (9212, Cell Signaling Technology, 1:1000), phospho‐EZH2 (Thr367) (PA5‐106225, Invitrogen, 1:500), EZH2 (ab186006, Abcam, 1:1000), H3K27me3 (Ab192985, Abcam, 1:1000), cytokeratin 5 (ab52635, Abcam, 1:1000), cytokeratin 8 (904 801, BioLegend, 1:1000), E‐cadherin (610 182, BD Transduction, 1:10 000) and vimentin (Ab28028, Abcam, 1:5000). GAPDH (2118, Cell Signaling Technology, 1:1000), β‐actin (Ab8226, Abcam, 1:1000), or vinculin (ab129002, Abcam, 1:25 000) were used as loading controls. Secondary antibody was either rabbit anti‐mouse IgG antibody (AP160P, EMD Millipore, 1:10 000) or goat anti‐rabbit IgG antibody (AP132P, EMD Millipore,1:5000–1:20 000).

### Live Cell Imaging

After 7 days of culture, 3D cultures in 30 mg mL^−1^ fibrin gels were transferred to an environmental chamber with humidified CO_2_ atmosphere at 37 °C. The cultures were imaged using inverted research microscope Eclipse Ti‐E with the Perfect Focus System equipped with PlanFluor 20×/0.75 air objective (Nikon). Images were taken at 10 min intervals for 1.5 h using transmitted light mode.

### RNA Sequencing and Data Analysis

Total RNA was extracted using RNeasy Mini kit (Qiagen) according to manufacturer's instructions and further purified with RNA Clean and Concentrator kit together with DNAse treatment (Zymo). Bulk RNA barcoding (BRB) sequencing method was then performed at the Functional Genomics Unit (University of Helsinki, Finland) as previously described.^[^ ^60^ ^]^ Briefly, RNA samples (10 ng) were barcoded, converted to cDNA, and amplified by PCR. The PCR products were pooled together, purified with 0.6X Agencourt AMPure XP Beads (Beckman Coulter) and tagmented using the Nextera kit (Illumina) to prepare sequencing libraries. Library quantity was assessed using a Qubit 2 fluorometer (Invitrogen) and the Qubit DNA HS Assay Kit (ThermoFisher Scientific) and library quality using the LabChip GXII Touch HT (PerkinElmer), with the DNA High Sensitivity Assay (PerkinElmer) and the DNA 5 K / RNA / Charge Variant Assay LabChip (PerkinElmer). The libraries were then sequenced using Illumina NextSeq 500 (Illumina), with a custom primer producing read 1 of 20 bp and read 2 (paired end) of 55 bp. Counts were analyzed with R in RStudio (v.4.2.1),^[^ ^119^, ^120^ ^]^ and differentially expressed genes (DEGs) between matrix‐cultured and suspension cells were determined using Bioconductor's edgeR package (v.3.8).^[^ ^121^ ^]^ When examining DEGs between matrices, suspension cells were excluded from the design matrix due to their variability. To analyze differences in gene expression profiles, this work used Gene Set Enrichment Analysis software (v.4.2.3)^[^ ^52^ ^]^ and Molecular Signatures Database (v7.5.1) (UC San Diego and Broad Institute) with gene sets containing 10–5000 genes. The results were visualized using Cytoscape (v.3.9.1)^[^ ^122^ ^]^ or Origin(Pro) (v.2022).

### Scanning Electron Microscopy Imaging

To prepare samples for scanning electron microscopy, 3D cell cultures were fixed and dried. First, gels were briefly rinsed with PBS. For primary fixation, proteins of the gel network and cells were crosslinked by incubating the gels in 2.5% glutaraldehyde – 2% paraformaldehyde in PBS overnight at 4 °C. For the control, suspension cells were embedded in 10 mg mL^−1^ fibrin and fixed immediately. Gels were then washed 3 × 5 min with PBS. For secondary fixation, cellular lipids were crosslinked with 0.2%–0.9% osmium tetroxide in PBS for 1–2 h at room temperature after which the gels were washed 3 × 5 min with water. Gels were dehydrated using ascending ethanol series of 30%, 50%, 70%, 90%, and 2 × 100%, for 10 min each. For the samples revealing spheroid cross‐sections, gels were frozen in liquid nitrogen and fractured. Ethanol was then exchanged for liquid CO_2_ in Bal‐Tec CPD 030 critical point dryer (Leica Microsystems) by changing the medium several times. To dry the sample, temperature was raised to 40 °C to bring CO_2_ above its critical point and the gaseous CO_2_ was removed. Alternatively, gels were freeze‐dried using tertiary butanol. Dried sample was cut into smaller pieces, mounted onto carbon adhesive, and coated with 4 nm of iridium in Leica EM ACE600 sputter coater (Leica Microsystems). The sample was then observed with field emission scanning electron microscope ZEISS Sigma VP with Gemini column (ZEISS) at 1.5 kV acceleration voltage. Cell‐free gels were prepared for scanning electron microscopy following similar protocol as for gels with cells, except that the cell‐free gels were fixed with 2.5% glutaraldehyde and osmium fixation was omitted.

### Bulk Rheology

Rheological properties of fibrin gels were measures using AR2000 (TA Instruments) stress‐controlled rheometer equipped with a Peltier system. Parallel‐plate geometry with a 20 mm stainless steel plate was used. Fibrin pre‐gel was loaded between the plates at 4 °C, after which the temperature was raised to 20 °C and fibrin was gelled for 30 min. To prevent solvent evaporation, the geometry was covered with a solvent trap and the solvent reservoir was filled with water to provide a humidified chamber. A strain sweep from 0.01% to 200% strain at 1 rad s^−1^ was performed, and shear storage modulus *G′* (elastic properties) and loss modulus *G* *″* (viscous properties) were determined from the average values in the linear viscoelastic range at 0.1%–1% strain. Young's modulus (*E*) values were calculated using the equation *E* = 2 × *G′* (1 + υ), where υ is Poisson's ratio. For elastic hydrogels and incompressible materials, Poisson's ratio is approximated to be 0.5.

### Magnetic Micro Rheology

A magnetic micro rheometer (micromanipulator type 1) was used to probe cell‐size‐scale micro rheology of fibrin gels, by exerting magnetic forces on magnetic poly(lactic acid) particles (Micromod #12‐02‐304; nominal diameter of 30 µm) within the gels and tracking the particles displacements.^[^ ^40^ ^]^ Non‐magnetic 6 µm polystyrene particles (Polysciences #15714‐5) were used to acquire a reference position for the displacements to eliminate the environmental noise during the measurements (i.e., displacements of the magnetic particles were subtracted from the ones of the reference particles). Each pre‐gel was mixed with the particles and loaded onto a custom‐made holder, after which the holder was coverslipped and maintained in a humidified chamber to prevent drying during gelation. Polymerized fibrin gels were then measured by simultaneously applying oscillatory, sinusoidal forces on the magnetic particles, at a frequency of 0.05 Hz relevant to cancer‐cell migration,^[^ ^123^ ^]^ and detecting the particles displacements. This work accounted for the alterations of the magnetic particles sizes by using a force‐per‐volume value of 2.64  × 10^5^ N m^−13^ (e.g., 3.73 nn for a 30‐µm‐diameter particle). The applied forces enabled the particles displacements that had an average amplitude from 150 nm to 1 µm corresponding to the linear viscoelasticity range. For each fibrin concentration, 4–5 gels were prepared and the particles movement in 3–6 locations within the gels was recorded. Absolute complex shear modulus |G*| and phase angle *θ* (in radians) were determined from these measurements, and the storage modulus *G′* was calculated from *G′* = cos(*θ*)  ×  |G*|.

### Image Analysis

To determine the proliferation degree of the cells, Ki67 score was calculated by counting at least 200 cells and dividing the number of Ki67 expressing cells by the total number of cells. To determine diameter of the spheroids, at least 15 spheroids for each fibrin gel concentration were measured with ImageJ. For evaluation of F‐actin area, the area of nuclei was subtracted from the area of nuclei/F‐actin overlay images (*n* = 4–5) in ImageJ.

### Statistical Analysis

The results are expressed as mean ± standard deviation of triplicate samples, unless otherwise noted. When comparing group means, ANOVA with Tukey HSD post‐hoc test was carried out in IBM SPSS 28. A *p*‐value below 0.05 was considered significant.

## Conflict of Interest

The authors declare no conflict of interest.

## Supporting information

Supporting Information

Supplemental Movie 1

## Data Availability

The RNA sequencing data have been deposited in the public NCBI GEO database and are accessible under accession code GSE228939.
