# Supplementary material for: Fibrin Stiffness Regulates Phenotypic Plasticity of Metastatic Breast Cancer Cells
Source: Adv Healthc Mater. 2023 Sep 21;12(31):2301137. doi: 10.1002/adhm.202301137 (PMC11469292; doi:10.1002/adhm.202301137)
Supplement: Supplementary file 1 — Supporting Information [file ADHM-12-2301137-s002.pdf]

ADVANCED
HEALTHCARE
MATERIALS

Supporting Information

for *Adv. Healthcare Mater.*, DOI 10.1002/adhm.202301137

Fibrin Stiffness Regulates Phenotypic Plasticity of Metastatic Breast Cancer Cells

Maria Heilala, Arttu Lehtonen, Ossi Arasalo, Aino Peura, Juho Pokki, Olli Ikkala, Nonappa, Juha Klefström and Pauliina M. Munne**

Supporting Information

Fibrin Stiffness Regulates Phenotypic Plasticity of Metastatic Breast Cancer Cells

Maria Heilala, Arttu Lehtonen, Ossi Arasalo, Aino Peura, Juho Pokki, Olli Ikkala, Nonappa, Juha Klefström, Pauliina M. Munne**

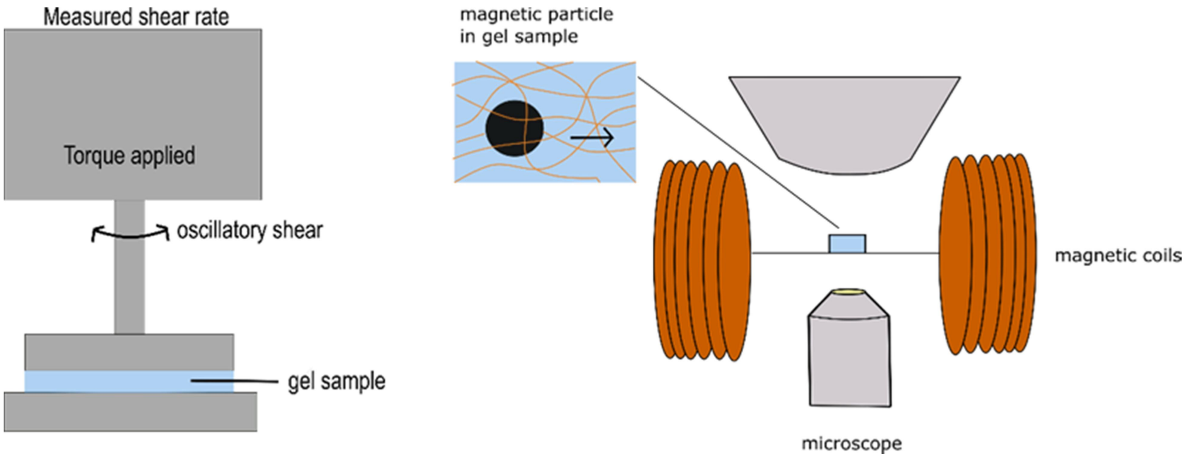

Figure S1. Schematic representation of setups used in bulk rheometry and magnetic microrheometry. In bulk rheometer, gel sample is loaded between two parallel plates and the motor applies torque to oscillate the upper plate. Shear rate of the sample is proportional to the global viscoelastic properties of the gel. In magnetic microrheometer, magnetic particles are embedded within the sample prior the gelation, after which the sample is transferred to a holder and allowed to gel. The gelled sample is placed under a microscope having magnetic coils integrated around the workspace. The magnetic particles are displaced using sinusoidal forces generated by the two magnetic coils. The movement of the magnetic particles is tracked from recorded videos. The particles displacement is inversely proportional to the local absolute shear modulus of the gels.

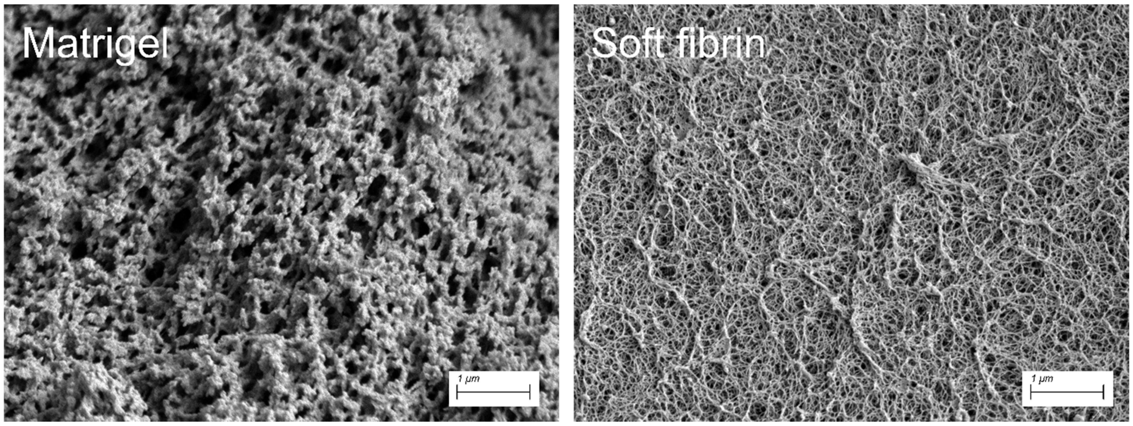

Figure S2. SEM images of matrix structure. Matrigel components were arranged into dense bundles, whereas soft fibrin was composed of thin long fibers. Scale bar is 1 µm.

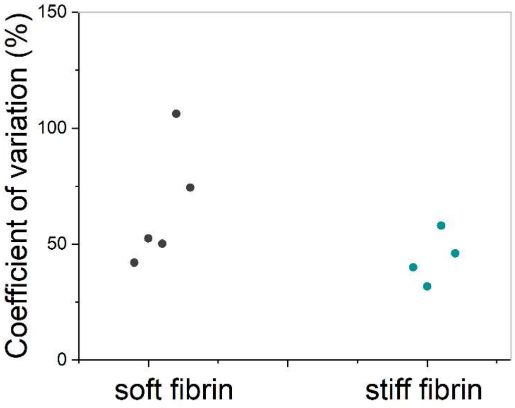

Figure S3. Coefficient of variation in fibrin gels measured in microrheology. Each point represents the variability of G* values in a single sample and thus intrasample heterogeneity.

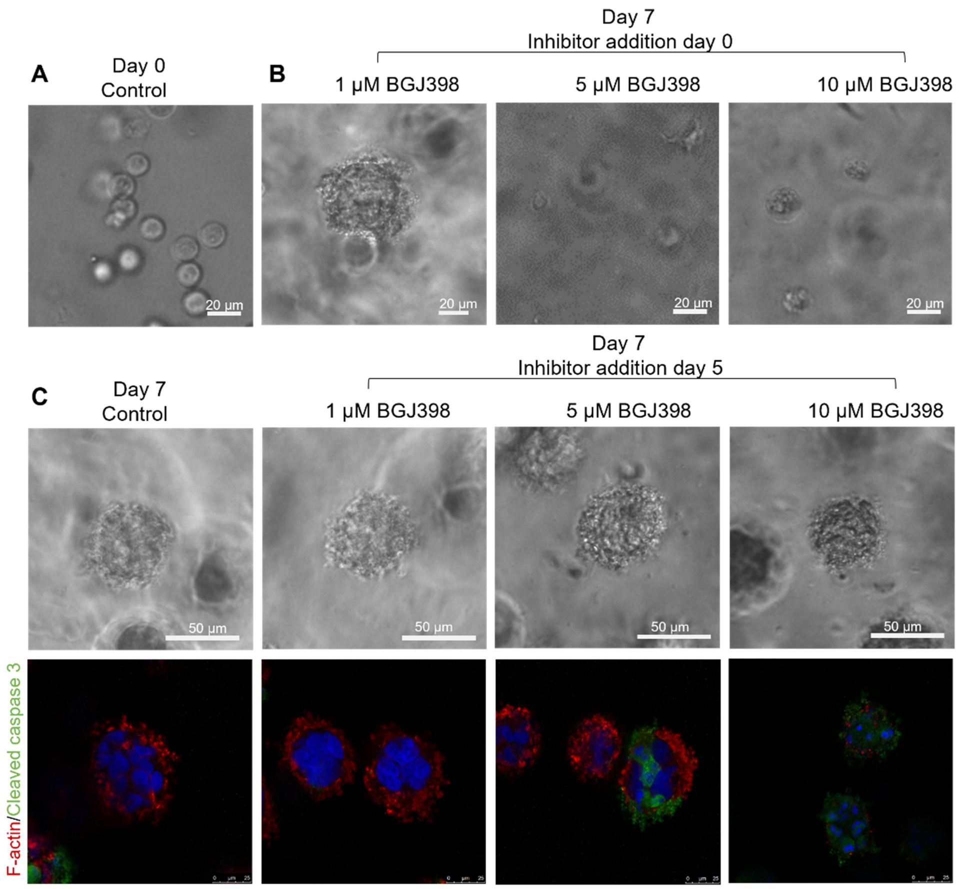

Figure S4. Inhibition of fibroblast growth factor receptor (FGFR) in cells cultured in stiff fibrin. DU4475 cells were seeded into stiff fibrin and cultured as described in experimental section, except 0-10 µM FGFR inhibitor infigratinib (BGJ398) was added to the medium on day 0 or 5. Control is cells without any inhibitor. Images were taken on day 7, unless otherwise specified. A) Light microscopy image of cells directly after gelation (day 0). B) Light microscopy image of cells cultured with various concentrations of inhibitor, added day 0. BGJ398 at 1 µM did not inhibit spheroid growth or protrusion formation, whereas 5 and 10 µM BGJ398 prevented cell growth (wrinkled cell fragments). C) Light microscopy images and immunofluorescent confocal images of cells stained for F-actin (red, cytoskeleton), nuclei (blue) and cleaved caspase 3 (green, cell death marker). Inhibitor was added after spheroid formation on day 5. The cells did not lose protrusions upon addition of 1-5 µM BGJ398. 10 µM BGJ398 lead to nuclear and cytoskeletal fragmentation, which indicates cell death. This is potentially due to non-specific effects at high inhibitor concentration. Scale bar is 20 or 50 µm in light microscopy images and 25 µm in immunofluorescent confocal images.

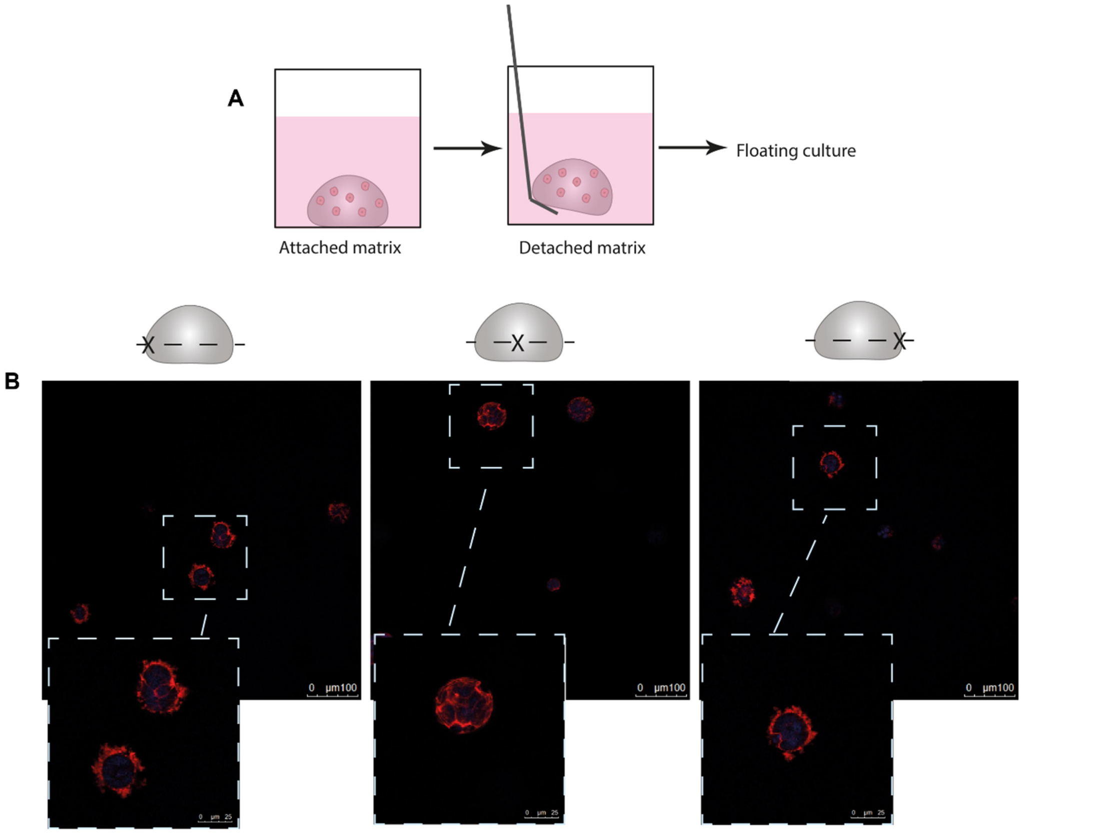

Figure S5. **Protrusion formation in stiff fibrin gel was not affected by floating culture**. A) Schematic of floating culture. After polymerization and addition of cell culture medium, stiff fibrin gel dome was carefully detached from the bottom of the culture vessel with a spatula. Cells were cultured in the floating matrix for seven days, after which cell distribution in different parts of the gel was evaluated. B) The cells were stained for nuclei (blue) and F-actin (red). Scale bar is 100 µm in the broad view and 25 µm in the inset.

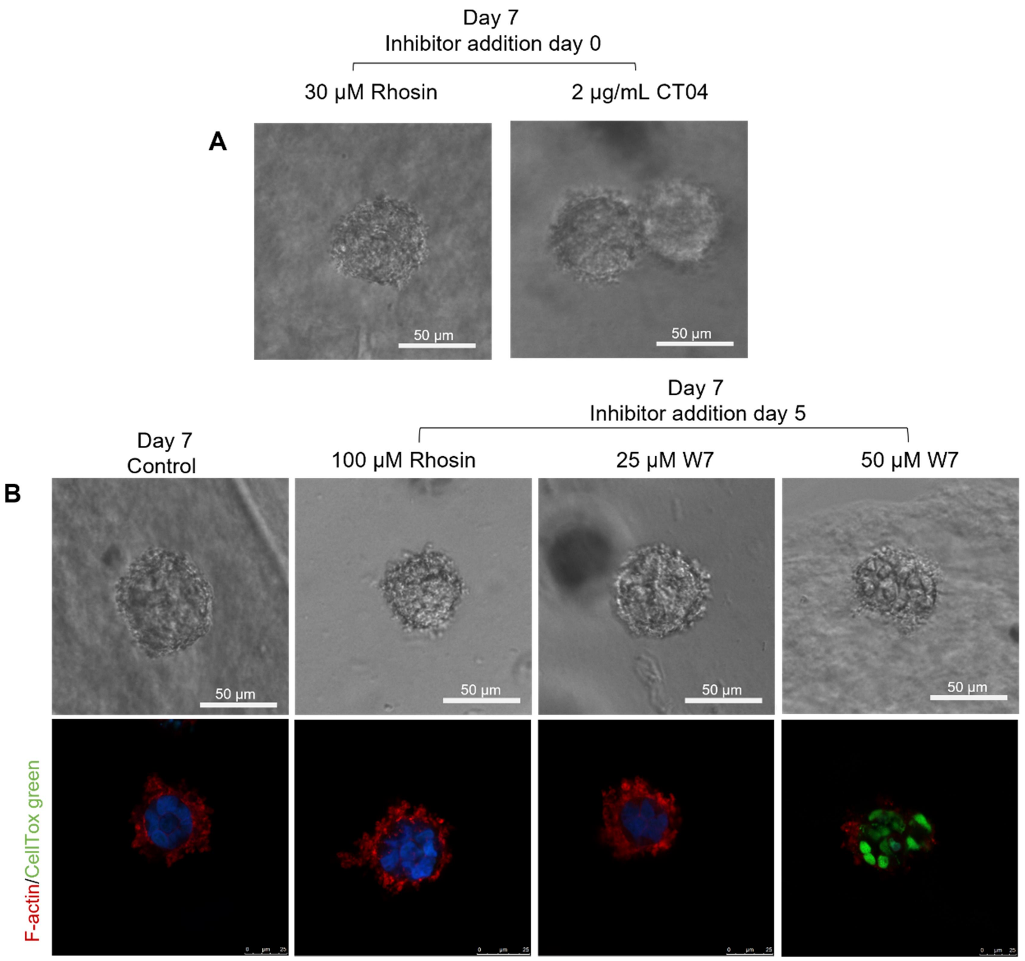

Figure S6. Inhibition of Rho and calmodulin-dependent actomyosin contractility in cells cultured in stiff fibrin. DU4475 cells were seeded into stiff fibrin and cultured as described in experimental section, except inhibitors targeting myosin light chain phosphorylation were added to the medium on day 0 or 5. Control is cells without any inhibitor. Images were taken on day 7. A) Light microscopy image of cells cultured with general Rho inhibitor CT04 (2 µg mL^{-1}) or specific RhoA inhibitor rhosin (30 µM), added day 0. Protrusion formation was not inhibited on day 7. B) Light microscopy images and immunofluorescent confocal images of cells stained for F-actin (red, cytoskeleton), nuclei (blue) and CellTox green (green, cell death marker). Indicated inhibitors were added after spheroid formation on day 5. Rhosin at 100 µM did not cause reversal of protrusions. Calmodulin inhibitor W7 at 25 µM did not affect protrusions but caused cell death at 50 µM. Scale bar is 50 µm in light microscopy images and 25 µm in immunofluorescent confocal images.

Table S1. Mechanical properties of fibrin gels measured in bulk rheology. The values were obtained from linear viscoelastic region of a strain sweep at 20 °C. Shear storage modulus *G´* represents the elastic and shear loss modulus *G´´* the viscous component of the gel. Phase angle varies from 0° to 90°, with values closer to zero indicating predominantly elastic properties. The Young's modulus E was calculated from $E = 2 \times G' (1 + \upsilon)$, where υ is Poisson's ratio that is assumed to be 0.5 for elastic hydrogels.

Gel	Young's modulus E (Pa)	Shear storage modulus G´ (Pa)	Shear loss modulus G´´ (Pa)	Phase angle (degrees)
**Soft fibrin (10 mg mL^{-1})**	171.0 ± 25.2	57.0 ± 8.4	4.2 ± 0.7	4.2 ± 0.3
**Stiff fibrin (30 mg mL^{-1})**	526.2 ± 86.4	175.4 ± 28.8	9.3 ± 0.7	3.1 ± 0.4

Table S2. Gene sets found to be enriched in comparisons between matrix-cultures. Annotation is the category used to group gene sets in Figure 4.

GENE SET NAME	GENE SET SIZE	ANNOTATION
REACTOME_STABILIZATION_OF_P53.V7.5.1.GRP	56	STRESS
REACTOME_CELLULAR_RESPONSE_TO_STARVATION.V7.5.1.GRP	155	STRESS
GOBP_CHAPERONE_MEDIATED_PROTEIN_FOLDING.V7.5.1.GRP	69	STRESS
HALLMARK_HYPOXIA.V7.5.1.GRP	193	STRESS
GOBP_POSITIVE_REGULATION_OF_EPITHELIAL_CELL_APOPTOTIC_PROCESS.V7.5.1.GRP	29	STRESS
GOBP_MEMBRANE_RAFT_ORGANIZATION.V7.5.1.GRP	23	CYTOSKELETON/MEMBRANE
GOBP_ACTIN_CROSSLINK_FORMATION.V7.5.1.GRP	13	CYTOSKELETON/MEMBRANE
GOBP_POSITIVE_REGULATION_OF_SUBSTRATE_ADHESION_DEPENDENT_CELL_SPREADING.V7.5.1.GRP	38	CYTOSKELETON/MEMBRANE
GOBP_REGULATION_OF_MICROTUBULE_BASED_MOVEMENT.V7.5.1.GRP	45	CYTOSKELETON/MEMBRANE
GOBP_ACTIN_FILAMENT_POLYMERIZATION.V7.5.1.GRP	152	CYTOSKELETON/MEMBRANE
GOBP_POSITIVE_REGULATION_OF_EPITHELIAL_TO_MESENCHYMAL_TRANSITION.V7.5.1.GRP	47	DIFFERENTIATION/EMT
HALLMARK_TNFA_SIGNALING_VIA_NFKB.V7.5.1.GRP	194	DIFFERENTIATION/EMT
WP_PLURIPOTENT_STEM_CELL_DIFFERENTIATION_PATHWAY.V7.5.1.GRP	42	DIFFERENTIATION/EMT
GOBP_POSITIVE_REGULATION_OF_WNT_SIGNALING_PATHWAY.V7.5.1.GRP	134	DIFFERENTIATION/EMT
PECE_MAMMARY_STEM_CELL_UP.V7.5.1.GRP	137	STEMNESS/PROLIFERATION
BHATTACHARYA_EMBRYONIC_STEM_CELL.V2022.1.HS.GRP	81	STEMNESS/PROLIFERATION
HALLMARK_E2F_TARGETS.V7.5.1.GRP	200	STEMNESS/PROLIFERATION
BENPORATH_PROLIFERATION.V2022.1.HS.GRP	147	STEMNESS/PROLIFERATION
REACTOME_G2_M_CHECKPOINTS.V7.5.1.GRP	165	STEMNESS/PROLIFERATION
REACTOME_EUKARYOTIC_TRANSLATION_INITIATION.V7.5.1.GRP	119	TRANSLATION/METABOLISM
REACTOME_TRANSLATION.V7.5.1.GRP	293	TRANSLATION/METABOLISM
GOBP_HISTONE_EXCHANGE.V7.5.1.GRP	20	TRANSLATION/METABOLISM
HALLMARK_MTORC1_SIGNALING.V7.5.1.GRP	199	TRANSLATION/METABOLISM
HALLMARK_GLYCOLYSIS.V7.5.1.GRP	197	TRANSLATION/METABOLISM
GOBP_POSITIVE_REGULATION_OF_GLYCOLYTIC_PROCESS.V7.5.1.GRP	19	TRANSLATION/METABOLISM
GOBP_EPIGENETIC_MAINTENANCE_OF_CHROMATIN_IN_TRANSCRIPTION_COMPETENT_CONFORMATION.V7.5.1.GRP	12	TRANSLATION/METABOLISM
GOBP_REGULATION_OF_HISTONE_PHOSPHORYLATION.V7.5.1.GRP	15	TRANSLATION/METABOLISM
